# Supplementary material for: Hook length of the bacterial flagellum is optimized for maximal stability of the flagellar bundle
Source: PLoS Biol. 2018 Sep 6;16(9):e2006989. doi: 10.1371/journal.pbio.2006989 (PMC6126814; doi:10.1371/journal.pbio.2006989)
Supplement: S1 Text — (DOCX) [file pbio.2006989.s001.docx]

**S1 Text**

**Observations from modeling of single-cell behavior**

The angular velocity *ψ* is related to the tangential velocity *v* and the radius *s* of the curvature of the cell trajectory as $\psi=\frac{v}{s} ,$making this parameter proportional to the cell velocity and inversely proportional to the radius of curvature. We observed that the angular velocity initially increases with hook-length. This corresponds to a decrease in the curvature radius of the trajectories, similar as observed before for sperm cells or other types of bacteria swimming near surfaces [1-5]. The angular velocity *ψ* decreases for longer trajectories, however, as a consequence of decreased run speed *v*. We also note that we were unable to reproduce the experimental observables of the short-hook (FliK_335_) che^−^ mutant by a single parameter couple (*ψ*,*λ*) in the context of our run-and-tumble model, which suggested that the movement of those mutants is not diffusive (S7 Fig). We suggest that the observed heterogeneous hook-length distribution of the FliK_335_ mutant (S3 Fig) explains the poor fit to a single parameter couple.

**S1 Text References**

1. Elgeti J, Kaupp UB, Gompper G. Hydrodynamics of sperm cells near surfaces. Biophys J. 2010;99(4):1018-26. Epub 2010/08/18. doi: 10.1016/j.bpj.2010.05.015. PubMed PMID: 20712984; PubMed Central PMCID: PMCPMC2920720.

2. Di Leonardo R, Dell'Arciprete D, Angelani L, Iebba V. Swimming with an image. Phys Rev Lett. 2011;106(3):038101. Epub 2011/03/17. doi: 10.1103/PhysRevLett.106.038101. PubMed PMID: 21405301.

3. Hu J, Wysocki A, Winkler RG, Gompper G. Physical Sensing of Surface Properties by Microswimmers--Directing Bacterial Motion via Wall Slip. Sci Rep. 2015;5:9586. Epub 2015/05/21. doi: 10.1038/srep09586. PubMed PMID: 25993019; PubMed Central PMCID: PMCPMC4438609.

4. Hu J, Yang M, Gompper G, Winkler RG. Modelling the mechanics and hydrodynamics of swimming E. coli. Soft Matter. 2015;11(40):7867-76. Epub 2015/08/11. doi: 10.1039/c5sm01678a. PubMed PMID: 26256240.

5. Lauga E, DiLuzio WR, Whitesides GM, Stone HA. Swimming in circles: motion of bacteria near solid boundaries. Biophys J. 2006;90(2):400-12. Epub 2005/10/22. doi: 10.1529/biophysj.105.069401. PubMed PMID: 16239332; PubMed Central PMCID: PMCPMC1367047.
