# Supplementary material for: Hook length of the bacterial flagellum is optimized for maximal stability of the flagellar bundle
Source: PLoS Biol. 2018 Sep 6;16(9):e2006989. doi: 10.1371/journal.pbio.2006989 (PMC6126814; doi:10.1371/journal.pbio.2006989)
Supplement: S2 Text — (DOCX) [file pbio.2006989.s002.docx]

**S2 Text**

**Derivation of the velocity autocorrelation function for the circular run-and-tumble model**

First, we start by defining the model. This is a true minimal model. We assume that bacteria can be regarded as point particles moving in two dimensions, along perfect circles, with a constant tangential speed. The angular velocity with which bacteria move along their circular path is $\nu$. Choosing the units such that the radius of the circular trajectory is unitary $s=1$, the trajectory of an individual bacterium can be parametrized with time $t$ as

(1) $\vec{r}\left( t \right)=\left( \cos\nu t,\sin\nu t \right)$

Correspondingly, the velocity of the bacterium $\vec{v}(t)=\frac{d\vec{r}}{dt}$ is

(2) $\vec{v}\left( t \right)=v\left( -\sin\nu t,\cos\nu t \right)$

From Eq. 2, we see that the initial velocity of the bacterium is

(3) $\vec{v}\left( 0 \right)=\left( 0,\nu\right).$

Next, we assume that after some time $\tau_{1}$ has passed, the bacterium will tumble and start moving along a different circle of the same radius and with the same angular velocity, i.e. the velocity will be changed by a phase shift $\phi_{i}\in\left[ 0,2\pi\right)$. We assume that tumbles can make the bacterium reorient towards any possible direction, and that the reorientations after each tumble are independent of one another (Markov property) i.e. $\phi_{i}$, $i\in\mathbb{N}$ are stochastic variables distributed as

(4) $P\left( \phi_{i} \right)=\frac{1}{2\pi}.$

We also assume that the intertumbling times $\tau_{i}$, $i\in\mathbb{N}$ are completely independent of each other, i.e. the intertumbling times are stochastic variables distributed as

(5) $P\left( \tau_{i} \right)=\lambda e^{-\lambda\tau_{i}},$

where the parameter $\lambda>0$ of the distribution is known as the tumbling rate.

The angular autocorrelation, defined as

(6) $g\left( t \right)=\frac{\vec{v}\left( 0 \right)\cdot\vec{v}\left( t \right)}{|\vec{v}\left( 0 \right)||\vec{v}\left( t \right)|},$

is a measure of the similarity of the initial velocity of the bacterium and its velocity at a later time $t$. Even though strictly speaking, the velocity of the bacterium at time $t$ is given by the expression

$$\vec{v}\left( t \right)=\nu\left( -\sin\left( \nu t+\sum_{i} \Theta\left( t-\tau_{i} \right)\phi_{i} \right),\cos\left( \nu t+\sum_{i} \Theta\left( t-\tau_{i} \right)\phi_{i} \right) \right),$$

where $\Theta\left( x \right)$ is the Heaviside function, due to the Markov property and statistical properties of the phase shifts $\phi_{i}$, and for the purposes of calculating the angular correlation this expression can be rewritten as

(7) $\vec{v}\left( t \right)=\nu\left( -\sin\left( \nu t+\xi\phi\right),\cos\left( \nu t+\xi\phi\right) \right),$

where $\phi=:\phi_{1}+\sum_{i=2} \Theta\left( t-\tau_{i} \right)\phi_{i}$ is an effective phase shift, distributed following Eq. 4 and

(8) $\xi:=\Theta\left( t-\tau_{1} \right)$

is a stochastic Boolean variable, $\xi\in\{0,1\}$.

The stochastic variable $\xi$ has a value of $\xi=0$ if the bacterium has not tumbled for the first time, and $\xi=1$ if it has tumbled at least once. From Eqs. 5 and 8, it is evident that the probability of $\xi=1$ is just the cumulative distribution function of $\tau_{i}$, that is

(9) $P\left( \xi=1 \right)=1-e^{-\lambda t}, \text{and}$

(10) $P\left( \xi=0 \right)=e^{-\lambda t}$

From Eqs. 3, 6, and 7 we have the following

$g\left( t \right)=\frac{\vec{v}\left( 0 \right)\cdot\vec{v}\left( t \right)}{|\vec{v}\left( 0 \right)||\vec{v}\left( t \right)|}$

$=\frac{\nu^{2} cos\left( \nu t+\xi\phi\right)}{\nu^{2}}$

$=\cos\left( \nu t+\xi\phi\right).$

The velocity autocorrelation function is defined as the expected value of the angular autocorrelation, $\left\langle g\left( t \right) \right\rangle$. Therefore, in this model, the velocity autocorrelation fucntion is given by

(11) $\left\langle g\left( t \right) \right\rangle=\left\langle\cos\left( \nu t+\xi\phi\right) \right\rangle,$

where the average is performed over all possible values of $\phi$ and $\xi$.

Due to the simplicity of the angular autocorrelation and probability distributions, the velocity autocorrelation function can be calculated analytically using trigonometric identities, Eqs. 4, 9, and 10, as follows

$\langle g(t)\rangle=\langle cos(\nu t+\xi\phi)\rangle$

$$=\int_{0}^{2\pi} \left[ \sum_{\xi=0}^{1} \cos\left( \nu t+\xi\phi\right)P\left( \xi\right) \right]P\left( \phi\right)\text{d}\phi$$

$$=\cos\left( \nu t \right)\int_{0}^{2\pi} \left[ \sum_{\xi=0}^{1} \cos\left( \xi\phi\right)P\left( \xi\right) \right]P\left( \phi\right)\text{d}\phi$$

$$-\sin\left( \nu t \right)\int_{0}^{2\pi} \left[ \sum_{\xi=0}^{1} \sin\left( \xi\phi\right)P\left( \xi\right) \right]P\left( \phi\right)\text{d}\phi$$

$$=\frac{\cos\left( \nu t \right)}{2\pi}\int_{0}^{2\pi} \left[ e^{-\lambda t}+\left( 1-e^{-\lambda t} \right)\cos\phi\right]\text{d}\phi$$

$$-\frac{\sin\left( \nu t \right)}{2\pi}\int_{0}^{2\pi} \left( 1-e^{-\lambda t} \right)\sin\phi\text{d}\phi$$

$$=\frac{\cos\left( \nu t \right)}{2\pi}\left[ e^{-\lambda t}\left. \phi\right|_{0}^{2\pi}+\left( 1-e^{-\lambda t} \right)\underset{=0}{\underbrace{\left. \sin\phi\right|_{0}^{2\pi}}} \right]$$

$$+\frac{\sin\left( \nu t \right)}{2\pi}\left( 1-e^{-\lambda t} \right)\underset{=0}{\underbrace{\left. \cos\phi\right|_{0}^{2\pi}}}$$

$$=\cos\left( \nu t \right)e^{-\lambda t}.$$

Therefore, in this simple model, the velocity autocorrelation function is given by

$$\left\langle g\left( t \right) \right\rangle=\cos\left( \nu t \right)e^{-\lambda t}.$$
