# Supplementary material for: Hook length of the bacterial flagellum is optimized for maximal stability of the flagellar bundle
Source: PLoS Biol. 2018 Sep 6;16(9):e2006989. doi: 10.1371/journal.pbio.2006989 (PMC6126814; doi:10.1371/journal.pbio.2006989)
Supplement: S3 Text — (DOCX) [file pbio.2006989.s003.docx]

**S3 Text**

**Effects of growth medium and temperature on single-cell behavior**

For experimental reasons, single cell tracking in quasi 2D was performed with cells grown in LB medium at 37 °C, whereas bacteria for the DDM and DFM experiments were grown in TB medium at 30 °C. While the overall motility behavior was remarkably similar between the experimental conditions (Fig 2, Fig 4, S9 Fig), we observed a substantial decrease in swimming speed for long hook mutants grown in LB at 37 °C. These differences may be attributed to increased flagella numbers per cell for bacteria grown in LB at 37 °C, which presumably exacerbate the increased bundle instability of the long hook mutants (S9 Fig).
