# Supplementary material for: Hook length of the bacterial flagellum is optimized for maximal stability of the flagellar bundle
Source: PLoS Biol. 2018 Sep 6;16(9):e2006989. doi: 10.1371/journal.pbio.2006989 (PMC6126814; doi:10.1371/journal.pbio.2006989)
Supplement: S1 Table — (DOCX) [file pbio.2006989.s004.docx]

**S1 Table:** List of *Salmonella* Typhimurium strains used in this study.

| **Strain** | **Genotype** | **Reference** |
| --- | --- | --- |
| EM2634 | *fliK*22363 (Δaa63-157) FliK310 | This study |
| EM2655 | *fliK*22364 (Δaa127-169) FliK335 | This study |
| TH9545 | *fliK*6436 (Δaa161-202) FliK363 | This study |
| TH10335 | *fliK*6619 (Δaa238-269) FliK373 | This study |
| TH9543 | *fliK*6434 (Δaa161-188) FliK377 | This study |
| TH437 | LT2 wild type FliK405 | John Roth |
| TH9798 | *fliK*6529 (*fliK*180'-YscP(321-381)-'181*fliK*) FliK466 | This study |
| TH8259 | *fliK*6128 (*fliK*140'-YscP(307-381)-'141*fliK*) FliK480 | This study |
| TH9795 | *fliK*6526(*fliK*140'-YscP(146-260)-'141*fliK*) FliK520 | This study |
| TH8258 | *fliK*6127(*fliK*140'-YscP(217-381)-'141*fliK*) FliK570 | This study |
| EM2799 | *fliK*22381 (*fliK*95'-YscP(138-353)-'96*fliK*) FliK620 | This study |
| EM2797 | *fliK*22379 (*fliK*95'-YscP(138-353)-'96*fliK* + *fliK*140'-YscP(307-381)-'141*fliK*) FliK695 | This study |
| EM2798 | *fliK*22380 (*fliK*95'-YscP(138-353)-'96*fliK* + *fliK*140'-YscP(146-260)-'141fliK) FliK735 | This study |
| EM2796 | *fliK*22378 (*fliK*95'-YscP(138-353)-'96*fliK* + *fliK*140'-YscP(217-381)-'141fliK) FliK785 | This study |
| TH12427 | Δ*fliHIJ*7367 | Lab stock |
| EM4083 | *fliK*22363 (Δaa63-157) Δ*hin*-5717::FCF (fliC-ON) FliK310 | This study |
| EM4082 | *fliK*22364 (Δaa127-169) Δ*hin*-5717::FCF (fliC-ON) FliK335 | This study |
| EM4081 | *fliK*6436 (Δaa161-202) Δ*hin*-5717::FCF (fliC-ON) FliK363 | This study |
| EM4084 | *fliK*6619 (Δaa238-269) Δ*hin*-5717::FCF (fliC-ON) FliK373 | This study |
| EM4078 | *fliK*6434 (Δaa161-188) Δ*hin*-5717::FCF (fliC-ON) FliK377 | This study |
| TH5861 | Δ*hin*-5717::FCF (fliC-ON) FliK405 | Lab stock |
| EM4077 | *fliK*6529 (*fliK*180'-YscP(321-381)-'181*fliK*) Δ*hin*-5717::FCF (fliC-ON) FliK466 | This study |
| EM4085 | *fliK*6128 (*fliK*140'-YscP(307-381)-'141*fliK*) Δ*hin*-5717::FCF (fliC-ON) FliK480 | This study |
| EM4080 | *fliK*6526(*fliK*140'-YscP(146-260)-'141*fliK*) Δ*hin*-5717::FCF (fliC-ON) FliK520 | This study |
| EM4079 | *fliK*6127(*fliK*140'-YscP(217-381)-'141*fliK*) Δ*hin*-5717::FCF (fliC-ON) FliK570 | This study |
| EM4087 | *fliK*22381 (*fliK*95'-YscP(138-353)-'96*fliK*) Δ*hin*-5717::FCF (fliC-ON) FliK620 | This study |
| EM4086 | *fliK*22379 (*fliK*95'-YscP(138-353)-'96*fliK* + *fliK*140'-YscP(307-381)-'141*fliK*) Δ*hin*-5717::FCF (fliC-ON) FliK695 | This study |
| EM4088 | *fliK*22380 (*fliK*95'-YscP(138-353)-'96*fliK* + *fliK*140'-YscP(146-260)-'141fliK) Δ*hin*-5717::FCF (fliC-ON) FliK735 | This study |
| EM4089 | *fliK*22378 (*fliK*95'-YscP(138-353)-'96*fliK* + *fliK*140'-YscP(217-381)-'141fliK) Δ*hin*-5717::FCF (fliC-ON) FliK785 | This study |
| EM4091 | Δ*fliHIJ*7367 Δ*hin*-5717::FCF (fliC-ON) | This study |
| EM3004 | *fliK*22363 (Δaa63-157) P*flhDC*5451::Tn*10d*Tc[del-25]/ pRG19 FliK310 | This study |
| EM3005 | *fliK*22364 (Δaa127-169) P*flhDC*5451::Tn*10d*Tc[del-25]/ pRG19 FliK335 | This study |
| EM3013 | *fliK*6436 (Δaa161-202) P*_flhDC_*_5451_::Tn*10d*Tc[del-25] ^a)^/ pRG19 FliK363 | This study |
| EM3012 | *fliK*6619 (Δaa238-269) P*_flhDC_*_5451_::Tn*10d*Tc[del-25] ^a)^/ pRG19 FliK373 | This study |
| EM3006 | *fliK*6434 (Δaa161-188) P*_flhDC_*_5451_::Tn*10d*Tc[del-25] ^a)^/ pRG19 FliK377 | This study |
| EM2999 | P*_flhDC_*_5451_::Tn*10d*Tc[del-25] ^a)^/ pRG19 FliK405 | This study |
| EM3003 | *fliK*6529 (*fliK*180'-YscP(321-381)-'181*fliK*) P*_flhDC_*_5451_::Tn*10d*Tc[del-25] ^a)^/ pRG19 FliK466 | This study |
| EM3002 | *fliK*6128 (*fliK*140'-YscP(307-381)-'141*fliK*) P*_flhDC_*_5451_::Tn*10d*Tc[del-25] ^a)^/ pRG19 FliK480 | This study |
| EM3001 | *fliK*6526(*fliK*140'-YscP(146-260)-'141*fliK*) P*_flhDC_*_5451_::Tn*10d*Tc[del-25] ^a)^/ pRG19 FliK520 | This study |
| EM3000 | *fliK*6127(*fliK*140'-YscP(217-381)-'141*fliK*) P*_flhDC_*_5451_::Tn*10d*Tc[del-25] ^a)^/ pRG19 FliK570 | This study |
| EM3007 | *fliK*22381 (*fliK*95'-YscP(138-353)-'96*fliK*) P*_flhDC_*_5451_::Tn*10d*Tc[del-25] ^a)^/ pRG19 FliK620 | This study |
| EM3009 | *fliK*22379 (*fliK*95'-YscP(138-353)-'96*fliK* + *fliK*140'-YscP(307-381)-'141*fliK*) P*_flhDC_*_5451_::Tn*10d*Tc[del-25] ^a)^/ pRG19 FliK695 | This study |
| EM3010 | *fliK*22380 (*fliK*95'-YscP(138-353)-'96*fliK* + *fliK*140'-YscP(146-260)-'141fliK) P*_flhDC_*_5451_::Tn*10d*Tc[del-25] ^a)^/ pRG19 FliK735 | This study |
| EM3008 | *fliK*22378 (*fliK*95'-YscP(138-353)-'96*fliK* + *fliK*140'-YscP(217-381)-'141fliK) P*_flhDC_*_5451_::Tn*10d*Tc[del-25] ^a)^/ pRG19 FliK785 | This study |
| EM3011 | Δ*fliHIJ*7367 P*_flhDC_*_5451_::Tn*10d*Tc[del-25] ^a)^/ pRG19 | This study |
| EM2912 | *fliK*22363 (Δaa63-157) Δ*cheY*22371::FKF FliK310 | This study |
| EM2911 | *fliK*22364 (Δaa127-169) Δ*cheY*22371::FKF FliK335 | This study |
| EM2913 | *fliK*6436 (Δaa161-202) Δ*cheY*22371::FKF FliK363 | This study |
| EM2914 | *fliK*6619 (Δaa238-269) Δ*cheY*22371::FKF FliK373 | This study |
| EM2915 | *fliK*6434 (Δaa161-188) Δ*cheY*22371::FKF FliK377 | This study |
| EM2916 | Δ*cheY*22371::FKF FliK405 | This study |
| EM2917 | *fliK*6529 (*fliK*180'-YscP(321-381)-'181*fliK*) Δ*cheY*22371::FKF FliK466 | This study |
| EM2918 | *fliK*6128 (*fliK*140'-YscP(307-381)-'141*fliK*) Δ*cheY*22371::FKF FliK480 | This study |
| EM2919 | *fliK*6526(*fliK*140'-YscP(146-260)-'141*fliK*) Δ*cheY*22371::FKF FliK520 | This study |
| EM2920 | *fliK*6127(*fliK*140'-YscP(217-381)-'141*fliK*) Δ*cheY*22371::FKF FliK570 | This study |
| EM2921 | *fliK*22381 (*fliK*95'-YscP(138-353)-'96*fliK*) Δ*cheY*22371::FKF Δ*cheY*22371::FKF FliK620 | This study |
| EM2922 | *fliK*22379 (*fliK*95'-YscP(138-353)-'96*fliK* + *fliK*140'-YscP(307-381)-'141*fliK*) Δ*cheY*22371::FKF FliK695 | This study |
| EM2923 | *fliK*22380 (*fliK*95'-YscP(138-353)-'96*fliK* + *fliK*140'-YscP(146-260)-'141fliK) Δ*cheY*22371::FKF FliK735 | This study |
| EM2924 | *fliK*22378 (*fliK*95'-YscP(138-353)-'96*fliK* + *fliK*140'-YscP(217-381)-'141fliK) Δ*cheY*22371::FKF FliK785 | This study |
| EM2925 | Δ*fliHIJ*7367 Δ*cheY*22371::FKF | This study |
| EM4649 | *fliK*22363 (Δaa63-157) Δ*invH-*sprB::FKF FliK310 | This study |
| EM3966 | *fliK*22364 (Δaa127-169) Δ*invH-*sprB::FKF FliK335 | This study |
| EM2020 | *fliK*6436 (Δaa161-202) Δ*invH-*sprB::FKF FliK363 | This study |
| EM2024 | *fliK*6619 (Δaa238-269) Δ*invH-*sprB::FKF FliK373 | This study |
| EM2019 | *fliK*6434 (Δaa161-188) Δ*invH-*sprB::FKF FliK377 | This study |
| TH16133 | Δ*invH-*sprB::FKF | Lab stock |
| EM4650 | *fliK*6529 (*fliK*180'-YscP(321-381)-'181*fliK*) Δ*invH-*sprB::FKF FliK466 | This study |
| EM2016 | *fliK*6128 (*fliK*140'-YscP(307-381)-'141*fliK*) Δ*invH-*sprB::FKF FliK480 | This study |
| EM2022 | *fliK*6526(*fliK*140'-YscP(146-260)-'141*fliK*) Δ*invH-*sprB::FKF FliK520 | This study |
| EM2015 | *fliK*6127(*fliK*140'-YscP(217-381)-'141*fliK*) Δ*invH-*sprB::FKF FliK570 | This study |
| EM3968 | *fliK*22381 (*fliK*95'-YscP(138-353)-'96*fliK*) Δ*invH-*sprB::FKF FliK620 | This study |
| EM4004 | *fliK*22379 (*fliK*95'-YscP(138-353)-'96*fliK* + *fliK*140'-YscP(307-381)-'141*fliK*) Δ*invH-*sprB::FKF FliK695 | This study |
| EM4005 | *fliK*22380 (*fliK*95'-YscP(138-353)-'96*fliK* + *fliK*140'-YscP(146-260)-'141fliK) Δ*invH-*sprB::FKF FliK735 | This study |
| EM3967 | *fliK*22378 (*fliK*95'-YscP(138-353)-'96*fliK* + *fliK*140'-YscP(217-381)-'141fliK) Δ*invH-*sprB::FKF FliK785 | This study |
| TH16562 | Δ*fliHIJ*7367 Δ*invH-*sprB::FKF | Lab stock |

^a)^ Expression of *flhDC* under control of the anhydrotetracycline-inducible promoter P*_tetA_*.
