# Supplementary figures and images for: Hook length of the bacterial flagellum is optimized for maximal stability of the flagellar bundle
Source: PLoS Biol. 2018 Sep 6;16(9):e2006989. doi: 10.1371/journal.pbio.2006989 (PMC6126814; doi:10.1371/journal.pbio.2006989)

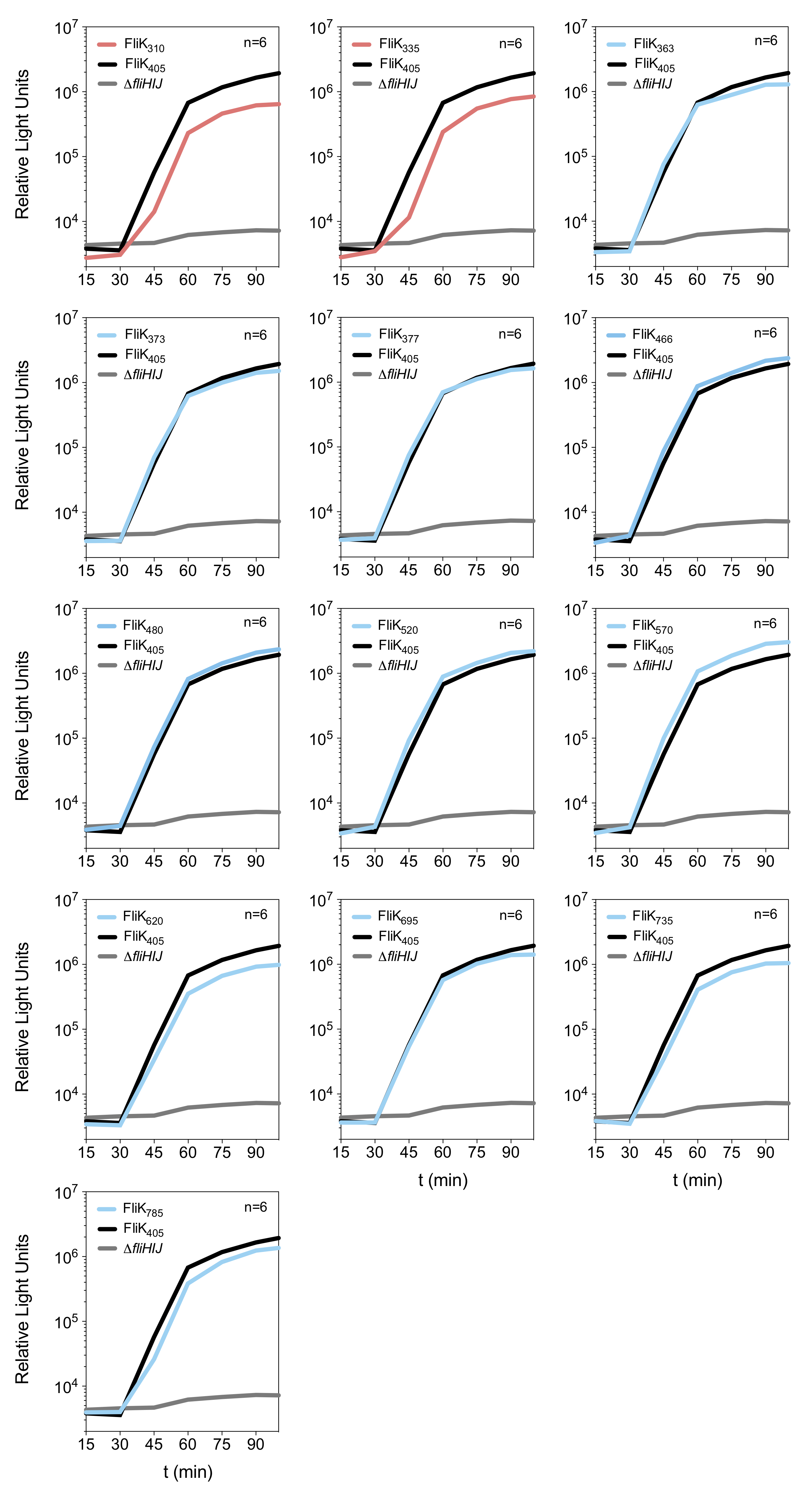

Supplement: S1 Fig — Expression of flagella synthesis was synchronized by induction of the PtetA-flhDC flagellar master regulatory operon. The switch in secretion specificity was monitored over time by measuring promoter activity of a Class 3 PmotA-luxCDABE reporter. Individual panels compare a hook-length mutant (FliK length indicated in aa), the wt (FliK405) and the negative control (ΔfliHIJ). Uncontrolled hook-length mutants are shown in red. aa, amino acid; wt, wild type. (PNG) [file pbio.2006989.s005.png]

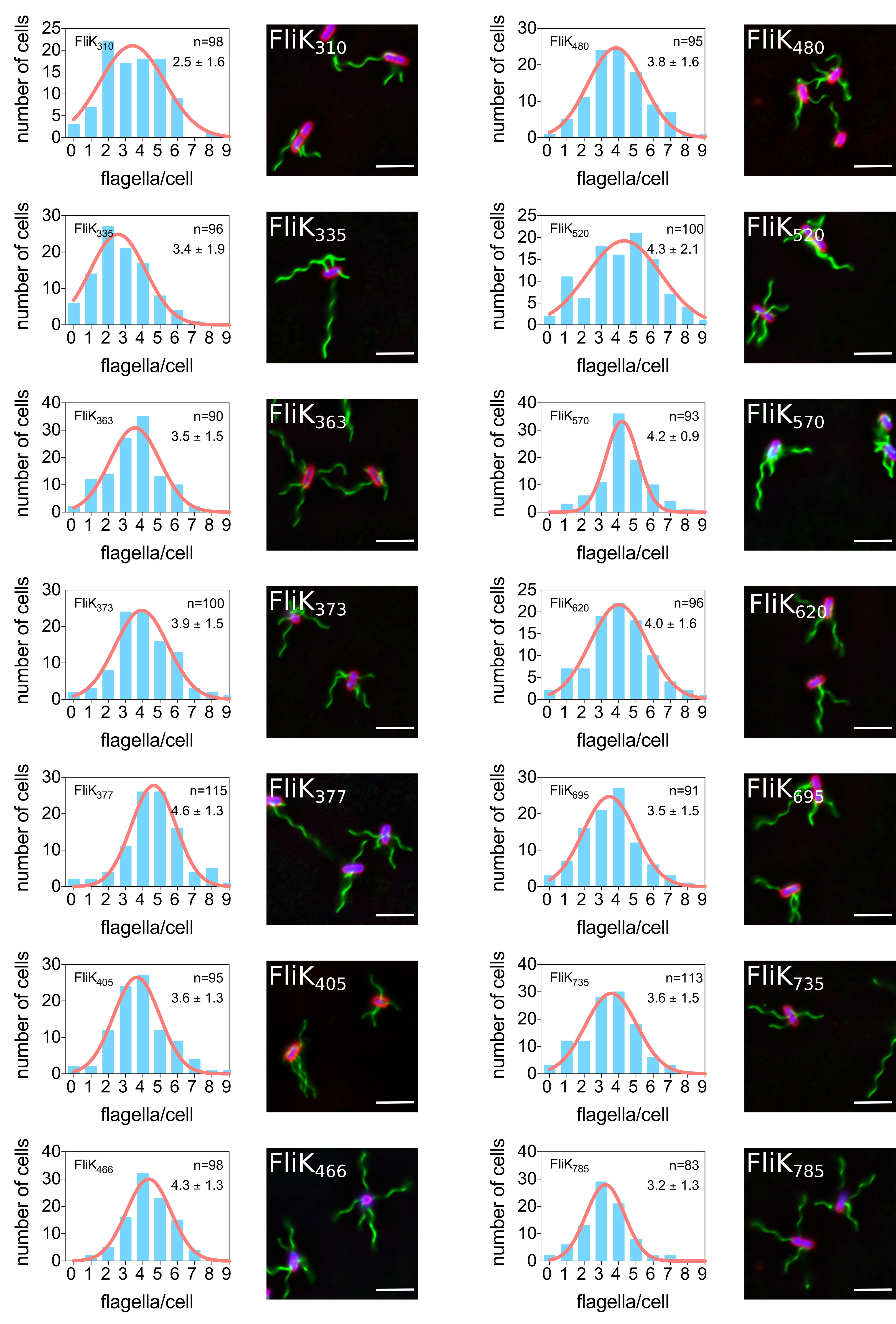

Supplement: S2 Fig — Quantification of the number of flagella of individual bacteria grown in LB at 37 °C (left panels). The flagellation pattern was determined by anti-flagellin immunostaining. Per mutant, the flagella per cell of all bacteria in 10 separate fields of view were counted manually. The average number of flagella per cell ± SD was calculated using Gaussian nonlinear regression analysis. Representative fluorescent microscopy images of anti-flagellin immunostaining (right panels). The flagellar filament was detected using anti-FliC immunostaining (green), membranes were stained using FM 4–64 (red), and DNA was stained using DAPI (blue). Scale bar = 5 μm. DAPI, 4′,6-diamidino-2-phenylindole; FM 4–64, N-(3-triethylammoniumpropyl)-4-(6-(4-(diethylamino) phenyl) hexatrienyl) pyridinium dibromide; LB, lysogeny broth. (PNG) [file pbio.2006989.s006.png]

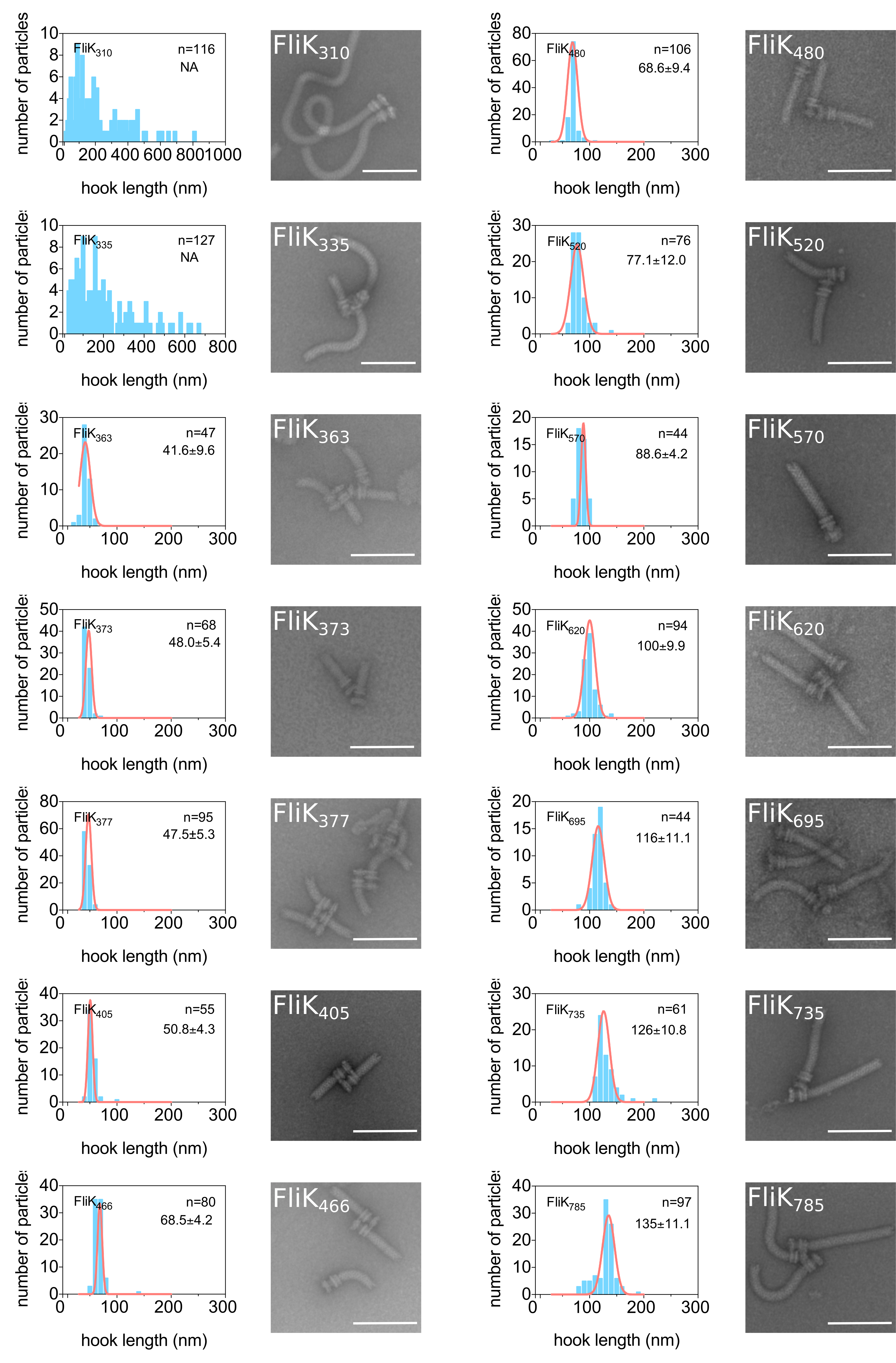

Supplement: S3 Fig — Histograms of hooks isolated from the FliK mutants (left panels). Hook-basal-body complexes were purified and imaged by negative staining electron microscopy. The average hook length ± SD was calculated using Gaussian nonlinear regression analysis. Representative electron micrograph images of purified hook-basal-body complexes (right panels). Scale bar = 100 nm. (PNG) [file pbio.2006989.s007.png]

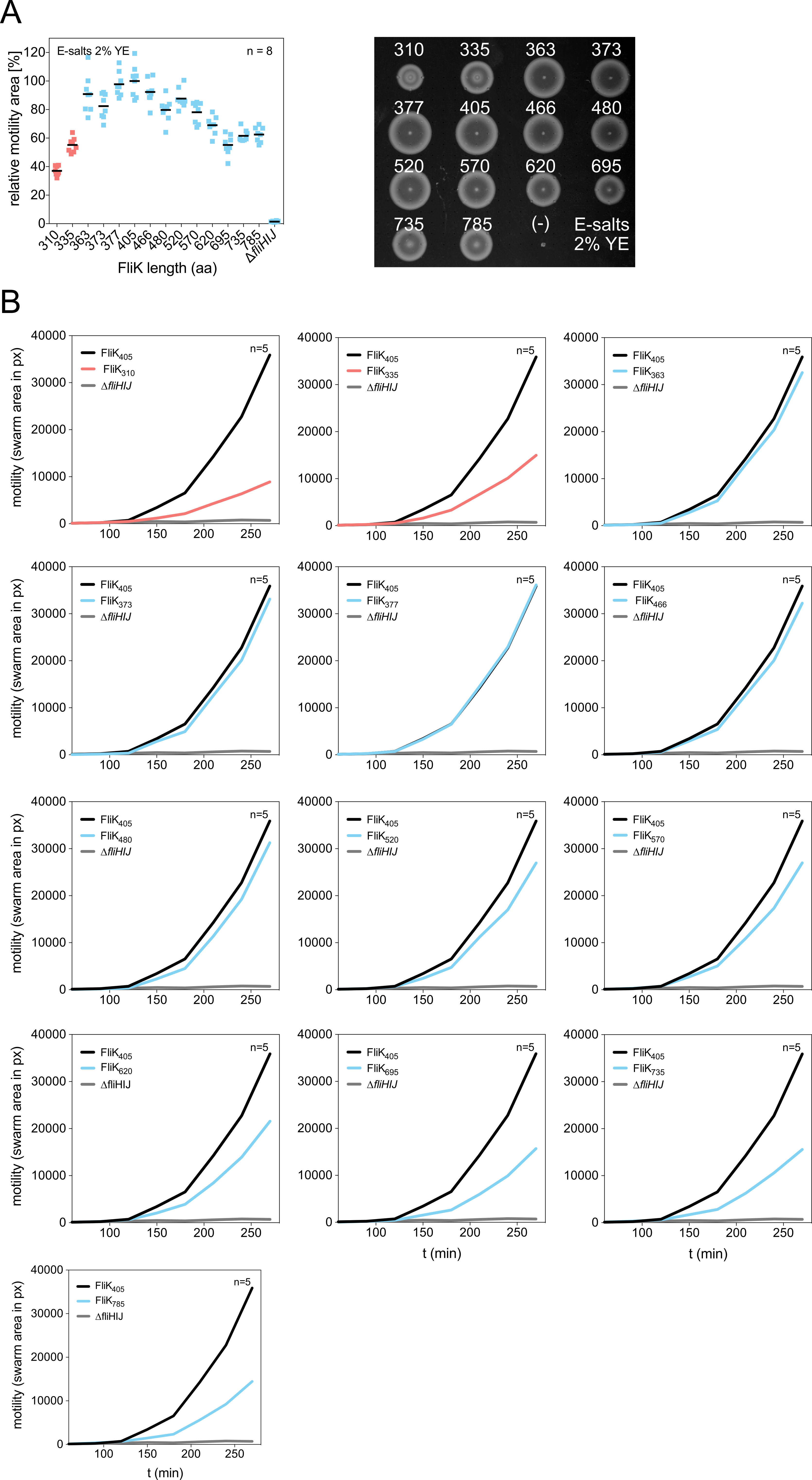

Supplement: S4 Fig — (A) Motility assay of the hook-length mutants in semisolid agar plates based on minimal medium containing 2% yeast extract (Vogel-Bonner medium E, 2% YE) and 0.3% agar. Quantification of the swimming motility assay (left panel) and representative semisolid agar plate (right panel). FliK length in amino acids is indicated; (−) indicates the nonflagellated control strain ΔfliHIJ. Motility halo size was measured using ImageJ and normalized to the wt (dots represent single data points; bars represent the mean; red dots represent the uncontrolled hook-length mutants). (B) Time course motility assay of the hook-length mutants in semisolid agar plates based on TB broth (1% tryptone and 0.5% NaCl) and containing 0.3% agar. Motility halo size was determined every 30 min and the average motility halo area of five biological replicates is shown. Uncontrolled hook-length mutants are shown in red. TB, tryptone broth; wt, wild type; YE, yeast extract. (PNG) [file pbio.2006989.s008.png]

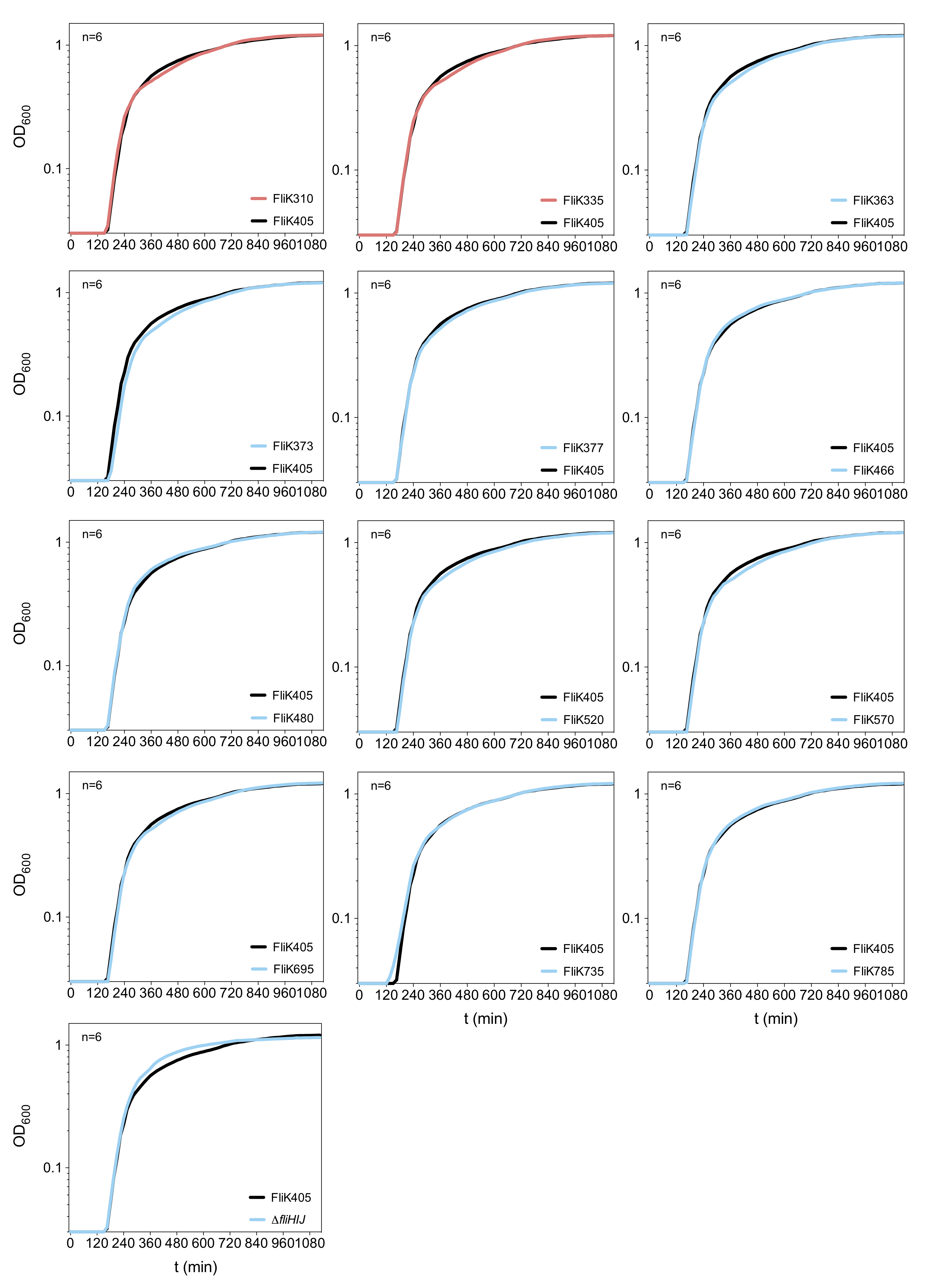

Supplement: S5 Fig — Bacteria were cultured in liquid LB medium and the OD600 was measured every 15 min. The average OD600 of six biological replicates is shown. Uncontrolled hook-length mutants are shown in red. LB, lysogeny broth. OD600, optical density at 600 nm. (PNG) [file pbio.2006989.s009.png]

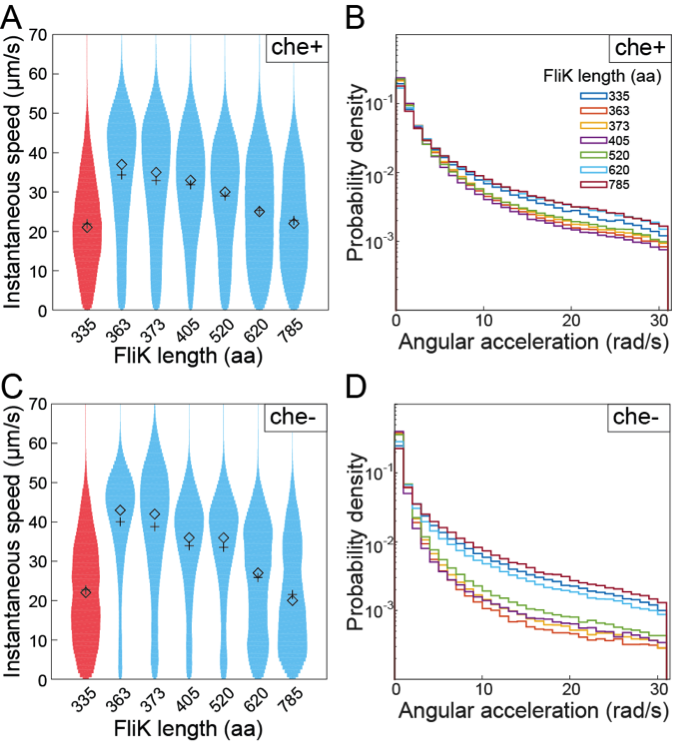

Supplement: S6 Fig — The distributions of motility parameters for different hook-length mutants. Distributions of (A) the instantaneous speeds and (B) the angular velocities pooled from all the trajectories from cells with a wt genetic background (che+). (C–D) The same distributions from smooth swimming mutants because of deletion of cheY (che−). Each distribution was calculated from more than 1,000 min of cumulative trajectory times pooled from three independent biological replicates, each containing more than 600 individual cells. Mean: +; median: ◊. wt, wild type. (PNG) [file pbio.2006989.s010.png]

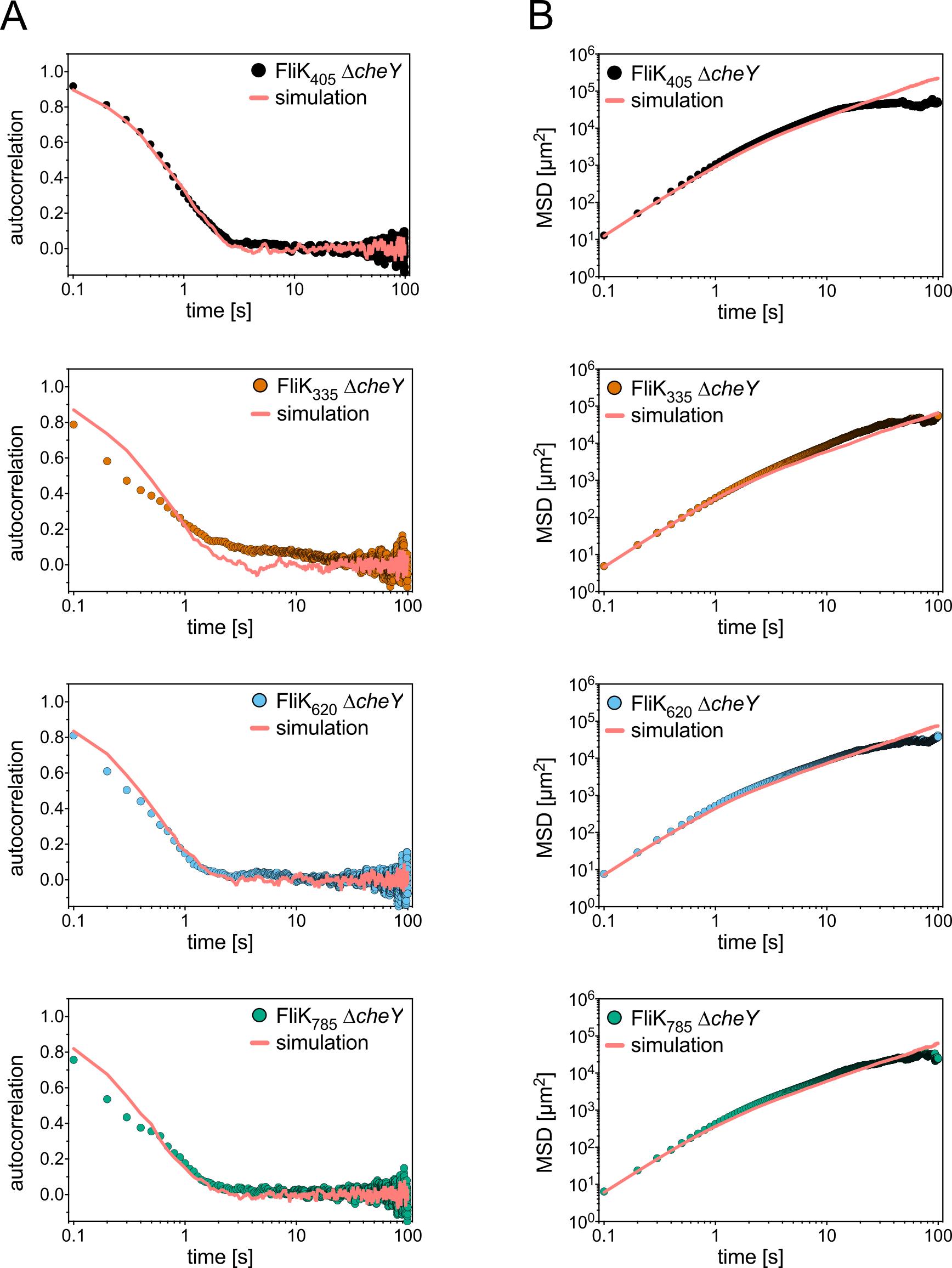

Supplement: S7 Fig — Observed orientational autocorrelation functions and circular run-and-tumble model autocorrelation function fits (A) and observed mean square displacements and circular run-and-tumble model mean square displacement fits (B) of chemotaxis deficient mutants (ΔcheY). Wt (FliK405) hook length is shown at the top. Hook length increases from top to bottom. The parameters of the model in each case are those reported in S2 Text. wt, wild type. (PNG) [file pbio.2006989.s011.png]

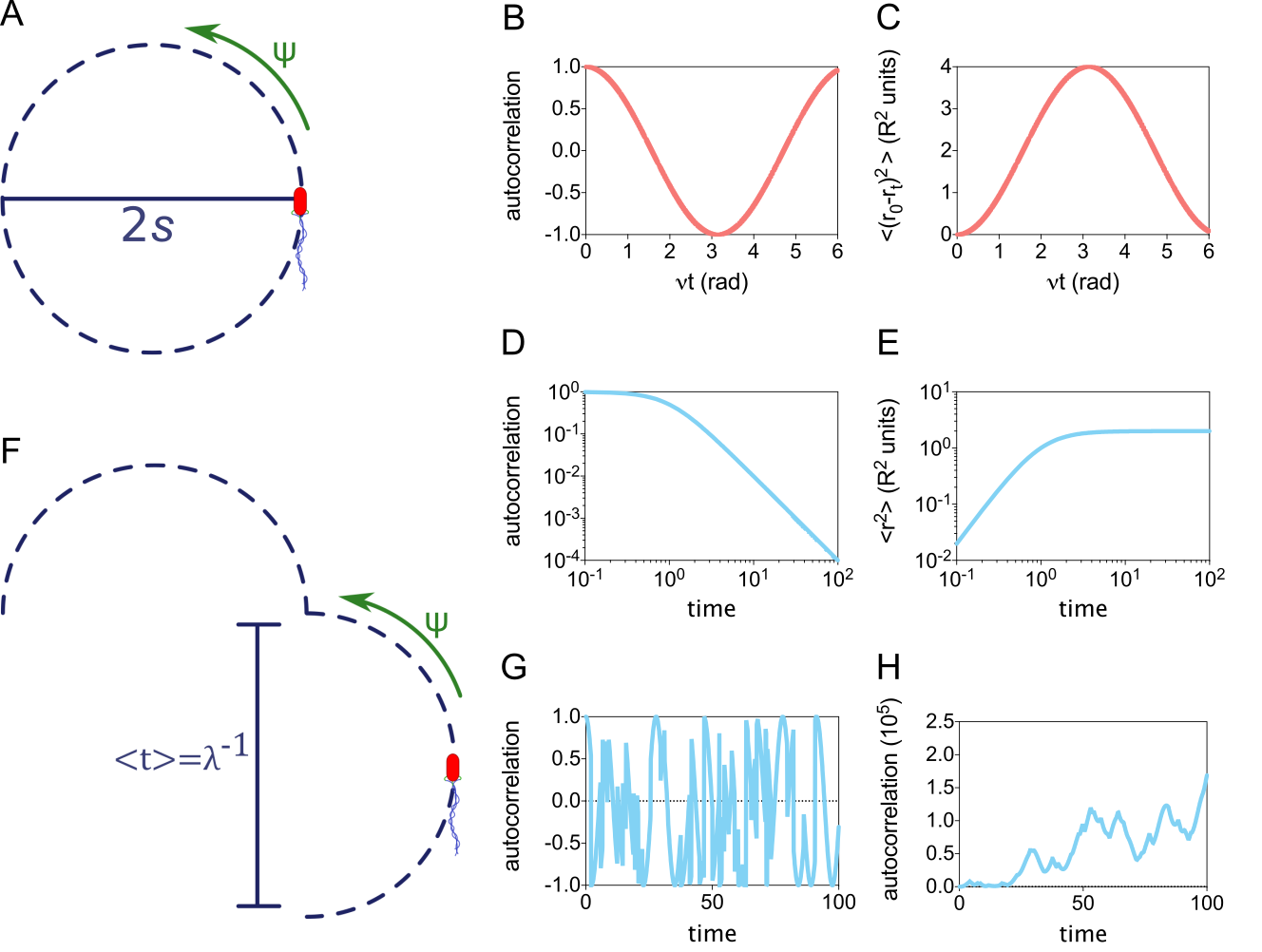

Supplement: S8 Fig — (A) In this model, individual cells are assumed to move on a circle of radius s with an angular velocity ψ. Under these conditions, the autocorrelation will be given by 〈c0→∙ct→〉=cos(ψt) (B) and the mean square displacement will be given by 〈r2〉 = 2s2[1 − cos(ψt)] (C). (D) Autocorrelation function and (E) mean square displacement under the assumption that the angular velocities are distributed among the population according to P(ψ). Shown are the observables when P(ψ) = λe−λψ. (F) Sample track of a virtual cell in the circular run-and-tumble model. The cell starts moving on a circle as in the uniform circular motion model; however, at exponentially distributed waiting times, the cell will reorient (tumble) and move along a new circular path. An example of autocorrelation of a single realization of the model is shown in panel G. An example of square displacement of a single realization of the model is shown in panel H. The value of the circular path radius s, necessary for the calculation of 〈r2〉, was obtained with the relation between the angular velocity ψ and tangential velocity v, ψ=vs. The value of ψ was obtained from the fitting of the model to the experimentally observed autocorrelation function, while the value of the tangential velocity v was obtained from the first data point of the observed mean square displacement 〈r2〉 of each strain by assuming ballistic movement at short times, i.e., 〈r2〉 ≈ 〈v2〉t2, therefore 〈v〉≈〈r2〉t2. (PNG) [file pbio.2006989.s012.png]

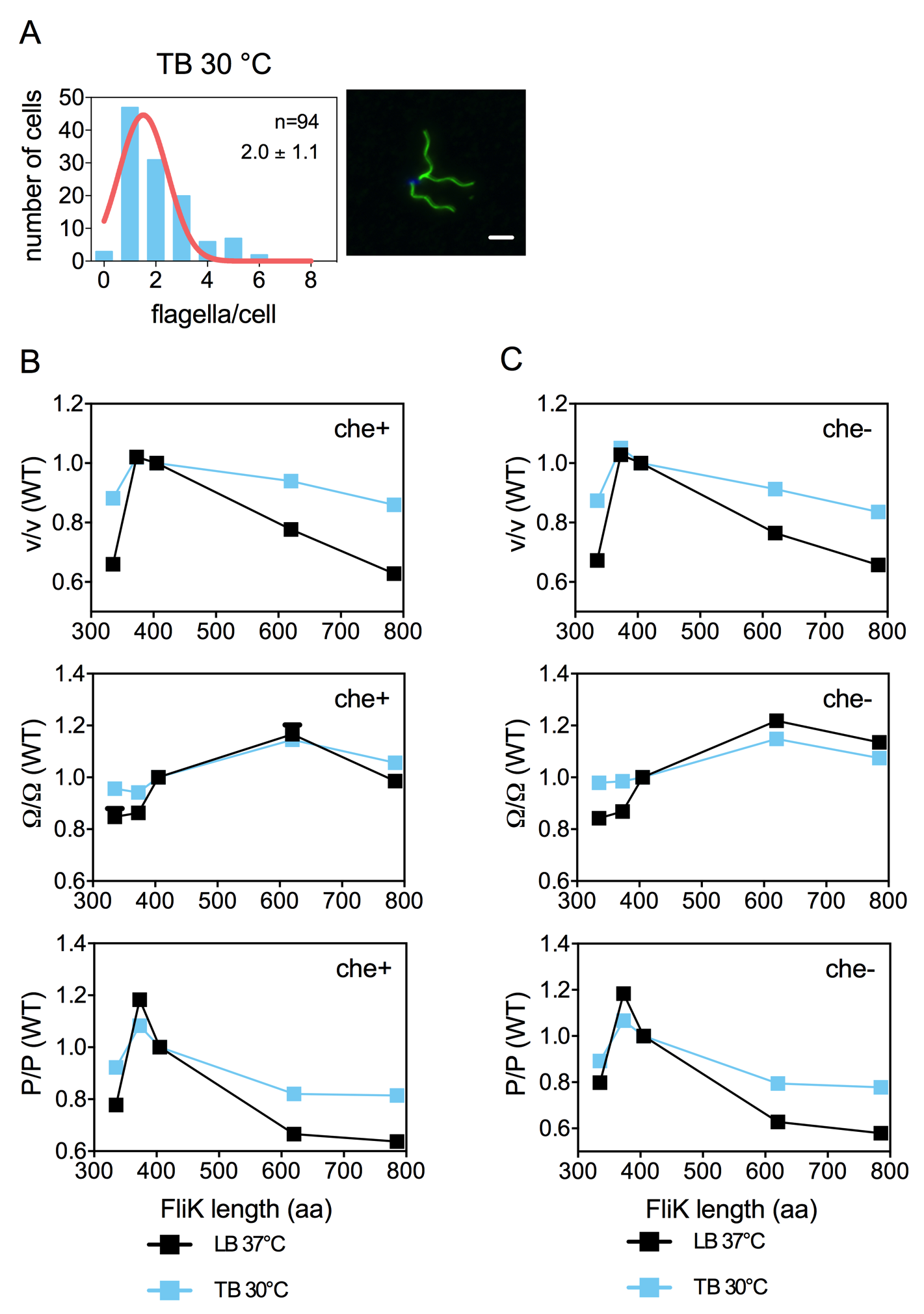

Supplement: S9 Fig — (A) Left: quantification of the number of flagella of individual bacteria grown in TB medium at 30 °C. The flagellation pattern was determined by anti-flagellin immunostaining. The average number of flagella per cell ± SD was calculated using Gaussian nonlinear regression analysis. Right: representative fluorescent microscopy images of anti-flagellin immunostaining. The flagellar filament was detected using anti-FliC immunostaining (green) and DNA was stained using DAPI (blue). (B) Measurement of swimming speed v, body rotational speed Ω, and processivity P = v / Ω relative to the wild type for hook-length mutants grown in LB at 37 °C and TB at 30 °C. (C) Measurement of swimming speed v, body rotational speed Ω, and processivity P = v / Ω relative to the wild type for chemotactic deficient (ΔcheY) hook-length mutants grown in LB at 37 °C and TB at 30 °C. DAPI, 4′,6-diamidino-2-phenylindole; LB, lysogeny broth; TB, tryptone broth. (PNG) [file pbio.2006989.s013.png]

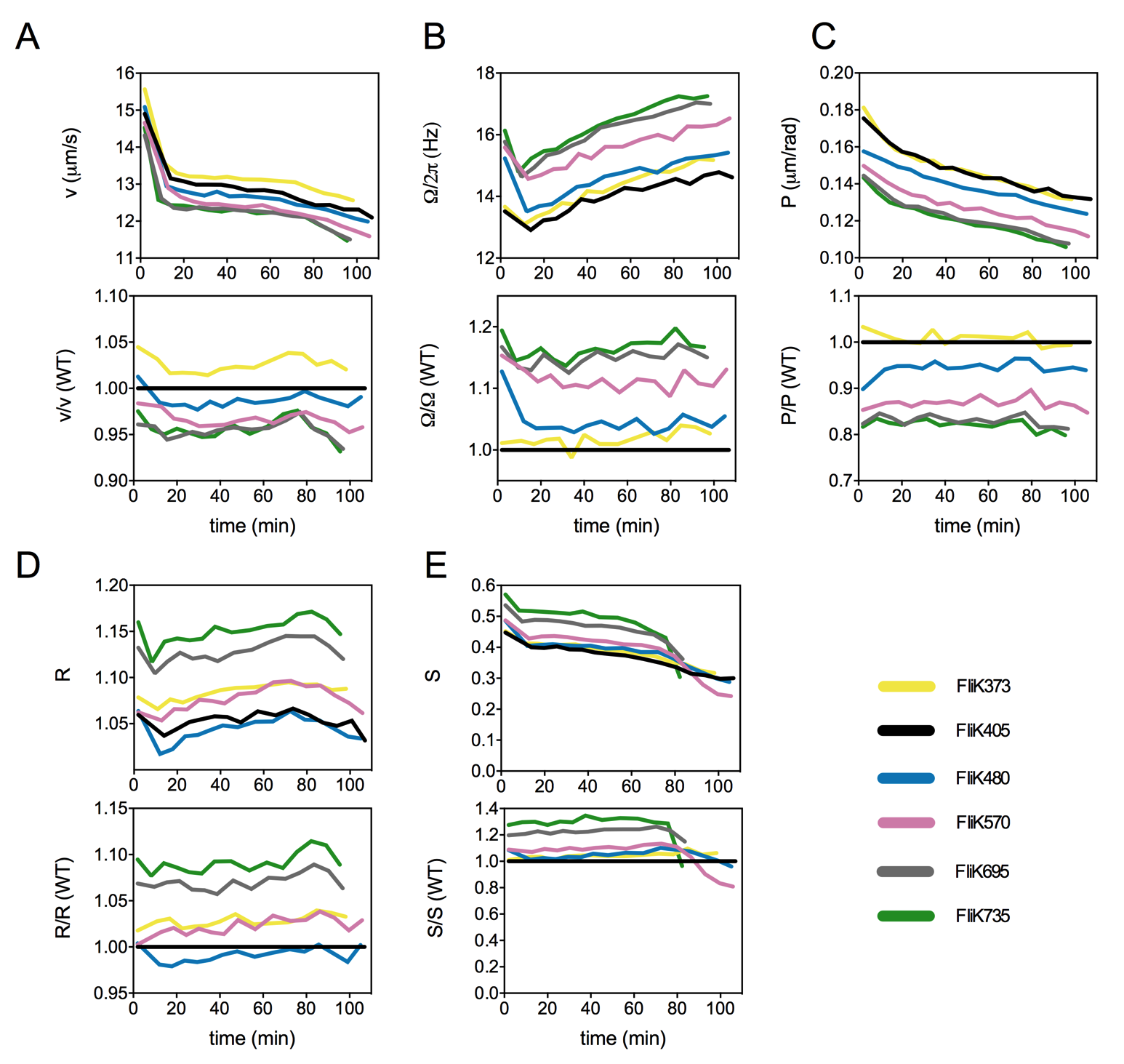

Supplement: S10 Fig — Absolute values (upper panels) and corresponding data normalized to the wt strain FliK405 (lower panels). (A) Swimming speed v, (B) body angular velocity Ω, (C) processivity P = v / Ω, (D) straightness of trajectory R, and (E) relative width S of the speed distribution P(v). Mean values presented in Fig 4 are obtained by averaging over the time window 10 min < t < 60 min. wt, wild type. (PNG) [file pbio.2006989.s014.png]

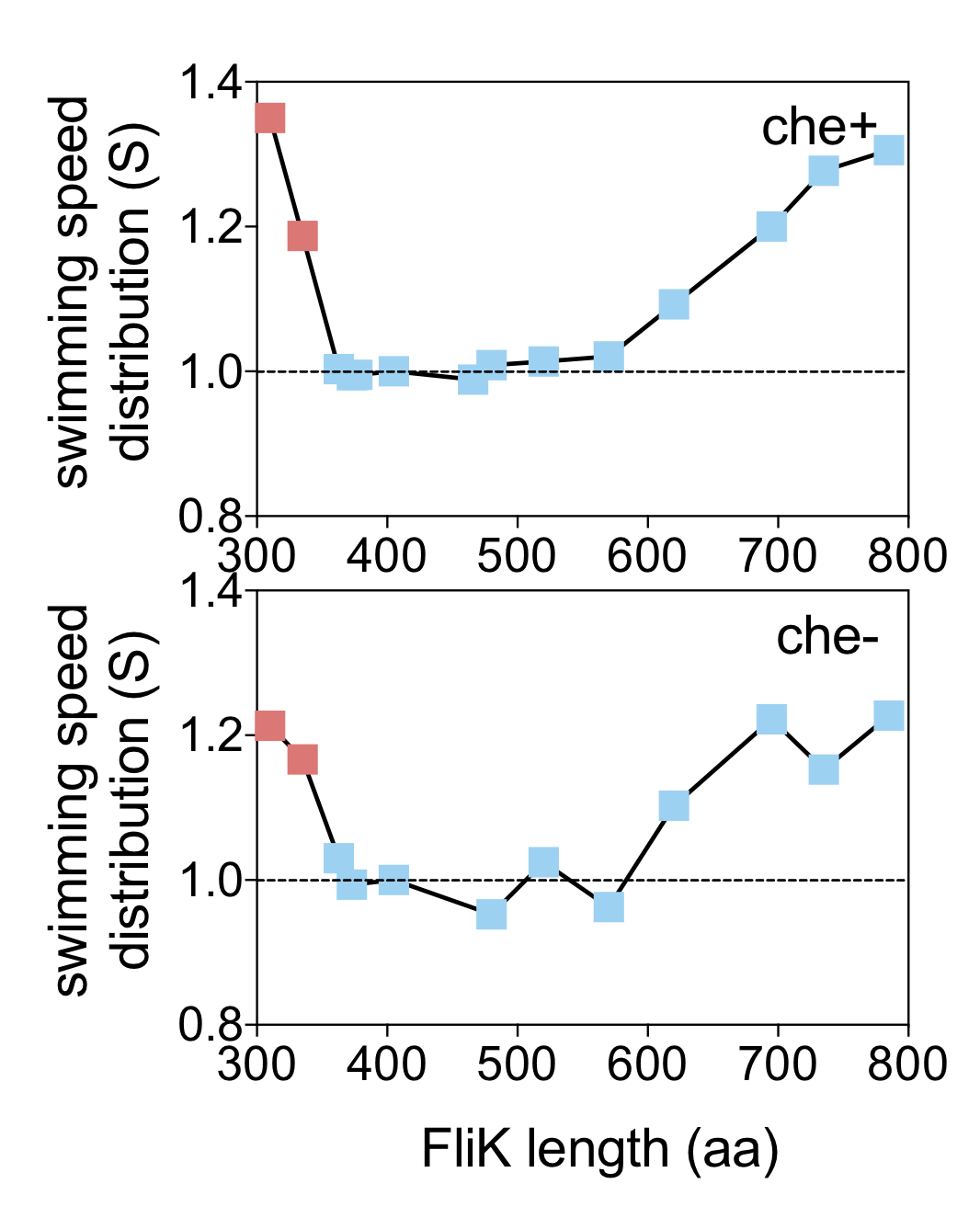

Supplement: S11 Fig — Upper panel: FliK mutants capable of chemotaxis; lower panel: smooth-swimming FliK mutants because of deletion of cheY. Data points represent the mean of an average of up to five experiments and were normalized to the wt. Uncontrolled hook-length mutants are shown in red. wt, wild type. (PNG) [file pbio.2006989.s015.png]

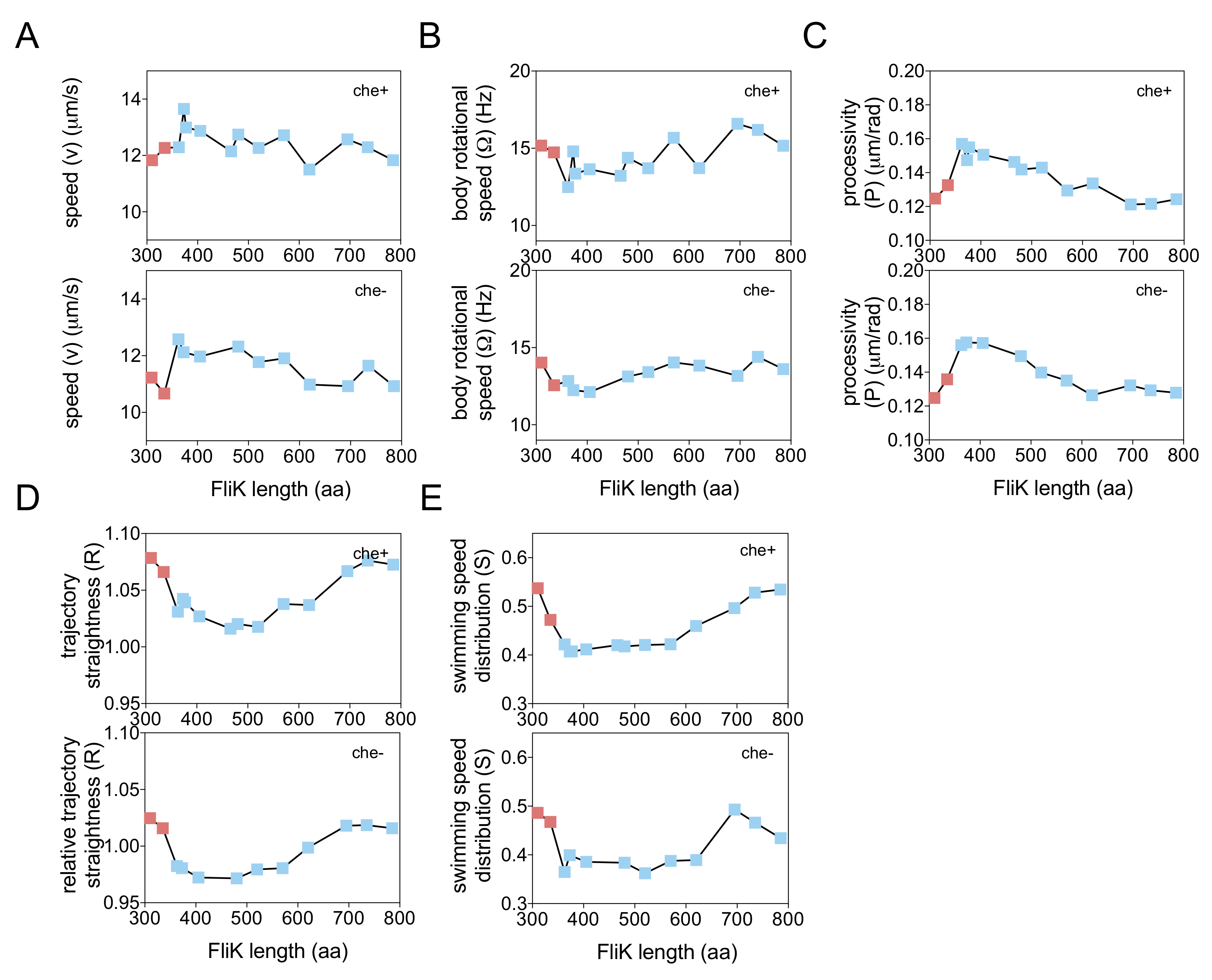

Supplement: S12 Fig — (A) Swimming speed v, (B) body rotational speed Ω, (C) processivity P = v / Ω, (D) straightness of trajectory R, and (E) relative width S of the speed distribution P(v). Upper panels: FliK mutants capable of chemotaxis; lower panels: smooth-swimming FliK mutants because of deletion of cheY. Data points represent the mean of an average of up to five experiments. Uncontrolled hook-length mutants are shown in red. (PNG) [file pbio.2006989.s016.png]
